# Evaluating Animal-Assisted Interventions: An Empirical Illustration of Differences between Outcome Measures

**DOI:** 10.3390/ani9090645

**Published:** 2019-09-03

**Authors:** Steffie van der Steen, Merel M.P. Heineman, Marloes J.A. Ernst

**Affiliations:** 1 Department of Pedagogical and Educational Sciences, University of Groningen, 9712 TS Groningen, The Netherlands; 2 Department of Psychology, Open University The Netherlands, Heerlen 6400, The Netherlands

**Keywords:** autism spectrum disorder, equine-assisted intervention, social skills, communication, parental report, observations, dose–response

## Abstract

**Simple Summary:**

This study compares and contrasts several outcome measures to assess the effect of an equine-assisted intervention for a child with Autism Spectrum Disorder. Before and after the equine-assisted sessions, we conducted a semi-structured interview with the participant’s parents, asked parents to fill out a general screening instrument separately, and observed the participant’s social and communication skills during five equine-assisted sessions. We found differences between the interview and questionnaire with regard to parents’ perceptions of aggression regulation and interacting with peers. Differences with regard to parental reports and observations were found for play development and anxiety. The observations provided a detailed view of the child’s development during the intervention, which yielded an interesting hypothesis in terms of the current dose–response discussion in AAI for children with Autism Spectrum Disorder.

**Abstract:**

Multiple authors have called for strong empirical evaluations to strengthen the foundation of Animal-Assisted Interventions. Carefully choosing the outcome measures of these studies is important, as choosing the wrong outcomes may lead to a failure to detect effects. The current study therefore compares and contrasts the use of several outcome measures, to assess the effect of an equine-assisted intervention for a child with Autism Spectrum Disorder: (1) a semi-structured interview with both parents, specifically designed for children with cognitive disabilities, (2) a general screening instrument filled out by both parents separately, which can be used to assess children’s psycho-social problems, and (3) systematic observations of social and communication skills during the equine-assisted sessions. All instruments indicated an improvement in the participant’s social and communication skills. We found differences between the interview and questionnaires with regard to parents’ perception of aggression regulation and interacting with peers. Differences with regard to parental reports and observations were found for play development and anxiety. The observations provided a detailed view of the child’s development during the intervention, which yielded an interesting hypothesis in terms of the current dose–response discussion in AAI for children with Autism Spectrum Disorder.

## 1. Introduction

Animal-Assisted Interventions (AAI) are structured interventions in health, education or social work, which incorporate animals to achieve therapeutic benefits [1]. As the field of AAI is growing, multiple authors have called for strong empirical evaluations to strengthen the foundation of these interventions [2,3,4,5,6]. Addressed considerations for improving scientific rigor are ensuring that sample sizes are large enough to obtain considerable statistical power, randomly assigning participants to conditions, and having follow-up measurements in place. The selection of outcome measures has been a topic of interest as well. For instance, Kruger and Serpell [3] mention a need for outcome measures that are relatively unbiased, that is, not sensitive to the expectancies or personal interests of the participants or informants. Serpell and colleagues [5] signal that carefully choosing the outcomes of a study is as important as the research design and statistical power. Choosing the wrong outcomes may lead to a failure to detect effects. While this may warrant the use of multiple outcome measures, Johnson, Odendaal and Meadows [7] warn for overburdening participants, especially when participants receive additional medical or mental health treatments. This may cause fatigue and can eventually lead to attrition. While studies on AAI have used several different outcome measures, no study has provided a detailed comparison of outcome measures to determine their applicability. The current study therefore set out to investigate the use of several measures (parental report and observations) to measure the effect of an equine-assisted intervention (AAI with horses) for a child with Autism Spectrum Disorder.

Children with Autism Spectrum Disorder (ASD) have been frequent recipients of AAI [2,8,9,10], especially dog- and equine-assisted interventions [8]. Besides a calming effect and a sense of support, animals may provide children with ASD a context to practice their social interaction and communication skills. These skills have been defined as one of the most promising outcomes of AAI for children with ASD [3,6], for which high effect sizes have been reported [2]. In an environment in which children feel safe, animals can provide feedback with regard to the effect of one’s own behavior on others and one’s perception of the behavior of others. In this way, interactions with animals affect self-awareness and social perceptions, and social interaction skills such as turn-taking can be practiced [3].

Researchers have chosen a variety of outcome measures to assess the influence of AAI on the social interaction and communication skills of children with ASD [6,8]. Most studies measured some form of verbal communication, such as verbal behavior directed to the therapist or animal [11,12,13,14], the frequency and duration of verbalizations [9], spontaneous or prompted verbalizations [15,16], productive and receptive language [17,18,19], parental report of communication skills [16,20,21,22], and language test scores [23]. With regard to social skills, studies have measured children’s non-verbal behavior directed to the professional or animal during the intervention [11,14], looking in the direction of the professional [9], spontaneous or prompted eye contact [12], seeking proximity, and petting the animal [15]. Some studies measured the ability to respond to social cues and sensory processing [17,24]. Other researchers focused on social motivation (i.e., motivation to engage in interactions) and volitional change [24]. Emotional expressions, positive social behaviors, positive affect and self-regulation have also been a topic of investigation [2,8,9,13,18,19,25,26], as well as sustained attention, focus and distractibility [12,24]. Lastly, studies have focused on children’s prosocial behavior [6,8], and adaptive reciprocal behavior in social interactions [12,17,22,25].

Typically, children’s social interaction and communication skills are studied in two ways: by asking parents or other informants through interviews or questionnaires, and through observations. Note that self-report instruments for children with ASD are not widely available, as their self-reflection skills may be particularly limited [27]. Researchers who did employ self-report measures in the AAI context used simple instruments with a few questions, and analyzed these in an exploratory way [27,28]. In addition, while children’s physiological responses to animals (salivary cortisol levels, skin conductance responses) are reported in the literature [28,29], these do not directly measure specific social and communication skills of children, and are therefore beyond the scope of this article.

Both parental reports and observations have their specific pitfalls. Informants, such as parents, are more likely to report positive results after AAI, because they expect children’s behavior to improve [27]. This form of response bias is well-documented in the social sciences literature [30,31]. Moreover, it is often difficult or unethical to conduct blind measurements in this context, that is, to keep parents uninformed about whether their child receives a particular intervention. While these issues can be overcome by adding reports of other informants such as teachers to the research design, three other issues are associated with the use of proxy reports. First, researchers in the AAI context often utilize validated standardized questionnaires or screening instruments that are quite broad [5]. Broad instruments, such as the Child Behavior Checklist, are not specifically designed to detect differences in behavior after an intervention, and as a result, may fail to detect such differences [32]. Second, researchers have found low to moderate agreement between multiple informants using standardized questionnaires on children’s (problem) behavior [33,34,35]. Reasons for discrepancies between raters are due to personality characteristics, the amount of time spent with the child, and the threshold that each informant has with regard to labeling children’s behavior as positive or negative [35]. Third, questionnaires and screening instruments filled out by informants provide a limited perspective on children’s interactions. That is, they do not inform researchers about *how* the child interacts, and only provide a measure of how the informant perceives the child’s behavior. Interactions between children and their social environment are, however, bidirectional, meaning that their quality depends on how the interaction between the child and the interaction partner flows in a natural context and in real time [36,37].

To evaluate children’s social interactions in real time, researchers may select observational methods. These methods are especially valuable when the quality of the interaction between child and animal is of interest, as emotions may be expressed differently in this context and the communication is largely nonverbal. Johnson and colleagues [7] argue that observations provide important clues about the context in which behavioral change takes place. At the same time, however, observations can also be biased. As in the case of parental report, “blind” observations are not always possible, especially when children’s behavior *during* the intervention is observed, providing the researcher with clues about the session number. By ensuring high levels of inter-rater agreement, this bias can be limited. However, inter-rater agreement may be difficult to achieve when complex constructs are observed [38]. Simple variables, such as the frequency of words or the length of utterances are easier to score reliably than higher-order variables, such as whether or not the child takes initiative, shows prosocial behavior or responds in an adequate way [36]. In addition, observational research is quite time-intensive, especially when observers first require training, and when multiple interactions are observed for each participant. This often results in small sample sizes, which may hinder generalization. Note, however, that small samples are not necessarily a weakness, especially when they provide a detailed view of children’s interactions by using multiple measures [39].

In sum, researchers in the AAI context have selected a variety of constructs and instruments to measure the communication and interaction skills of children with ASD. While the exact nature of their measures and instruments differs, the most commonly used methods are proxy reports (interviews and questionnaires) and observations. Both methods have advantages and disadvantages. The current study therefore compares the results of several measures in the context of an equine-assisted intervention for a child with ASD. The goal is to compare and contrast the measures, and thereby to assess their applicability. The following research questions are central: (1) Which changes in social and communication skills are reported by the parents, using a specific and a more general instrument? Do the results of these two instruments align, and do parents agree in their assessment? (2) Which changes in social and communication skills of the participant can be detected based on researchers’ systematic observations of behavior during the equine-assisted intervention? (3) Do the standardized questionnaire scores align with the behavioral changes that are observed during the therapy sessions?

We used a single-subject design in which the following measures were taken before and after an equine-assisted intervention: (1) a semi-structured interview with both parents specifically designed to measure social-emotional skills of children with cognitive disabilities [40], (2) a general screening instrument that can be used to assess psycho-social problems and strengths of children [41], and (3) systematic observations of the participant’s social and communication skills during the equine-assisted sessions.

## 2. Materials and Methods

### 2.1. Participant Information

For this study, an eight-year-old girl was chosen as a case and was offered five weekly equine-assisted sessions. By means of observations and parental questionnaires, changes in her social and communicative behavior were registered and analyzed. At the time of the therapy sessions, the participant was eight years and seven months old. Her brother, six years old at the time of the study, was present during all sessions, as well as their mother (age 41) and father (age 45). Both parents were college-educated. The mother worked in the field of arts/history, and the father worked in information technology. The family lived in an urban area in the south of the Netherlands.

The participant was enrolled in preschool at the age of three, and was transferred to a specialized child care facility at the age of four, where she was diagnosed with an Autistic Disorder using the Diagnostic and Statistical Manual of Mental Disorders-IV-TR diagnostic criteria [42]. At five years old, the participant was enrolled in a school for special education that provides smaller class sizes and specialized support for students with special educational needs. The participant completed the Dutch Wechsler Preschool and Primary Scale of Intelligence-III [43] at school when she was six years and seven months old. Her full-scale IQ was estimated at a low-average level of 77, 95% CI [70,80]. Her performance IQ and verbal IQ were both estimated at a low-average level of 81 and 89 respectively. She scored 55 on the processing speed quotient, 95% CI [52,77].

The parents reported that the participant experienced excessive anxiety when separated from her mother. Although the participant was able to verbalize her thoughts, she also had trouble to engage in reciprocal conversations and her turn-taking skills were limited. Repetitive and excessive questioning frequently occurred, and she often abruptly changed the subject of the conversation to (irrelevant) topics that were familiar to her, such as her favorite movies. In the past, the participant had received interventions that were mostly focused on mutual play and sensory regulation. No additional interventions were offered to the participant during this study.

### 2.2. Procedure

The mother of the participant responded to an online advertisement announcing a case study on an equine-assisted intervention for children 8–15 years old with ASD. After this initial contact, a researcher visited the family and explained the procedure of the study. The parents signed a written consent form during this meeting, and the researcher explained the purpose and duration of the study, that participation was voluntary, that the data would be stored and processed anonymously, and that they could withdraw from the study at any moment in time. The professional providing the equine-assisted intervention was present during this meeting as well and signed a written consent form and confidentiality form. Next, important focus areas of the treatment were discussed: separation anxiety, turn-taking skills, and emotions. During a second meeting the researcher met with the participant, administered the Scale for Emotional Development-Revised (SED-R; [40]) to both parents, and asked the parents to fill out the Strengths and Difficulties Questionnaire (SDQ; [41,44]).

After these two meetings, five weekly therapy sessions of 90–120 min were scheduled in a nearby rural wooded area. The horse involved in the sessions was a female Danish breed, a Knabstrupper, and she was 15 years old. The well-being of the horse was ensured by an assistant, whose only responsibility was to ensure the horse’s safety, enough rest, and access to food and water during the therapy sessions.

Each session was guided by the same professional, who was trained as an educator and as a New Trails Learning System equine-specialist [45]. In this program, the participant engages in relaxation exercises on the back of the horse (sensory work), therapeutic horseback riding (longlining) and rule-based interaction games. Throughout the sessions, the horse facilitates a connection between child and therapist. When the child interacts with the horse, such as stroking or talking to the horse, the therapist uses this to engage in interaction with the child. When the child engages in relaxation exercises on the back of the horse, the body temperature of the horse and calm breathing of the horse soothes the child. During horseback riding, the child sees the world from a different angle, and these new impressions stimulate conversation with the therapist, parents, and/or volunteers.

During horseback riding, the horse was guided by the professional using long reins. The long reins serves two purposes here. First, it gives the impression that the child leads the horse and decides on the direction and pace, which could ultimately boost the child’s self-confidence. Second, long reins enable the horse to move naturally and rhythmically, which is comfortable for both the horse and the rider. Two volunteers were present on both sides of the horse to ensure the safety of the participant. Meanwhile, the volunteers and professional stimulated the conversation skills of the participant by talking to her and the other family members. During the relaxation exercises, the participant sat on the back of the horse unsaddled while the horse was standing still, with two volunteers on both sides of the horse to ensure safety. The participant was then guided by the professional into a lying position on the back of the horse. Apart from activities in which the horse was actively involved, a number of rule-based interaction games were played in presence of the horse, in which the participant, professional, family members and volunteers participated. In these games, such as hide and seek and playing tag, adequate turn-taking is important. The professional explained these games to the participant and during the game the participant was guided by the professional, volunteers and family members, for example by indicating it was her turn to chase the others.

The professional and volunteers closely observed and followed the participant during the sessions. For example, during conversations they would respond to the participant’s initiations, or initially steer the conversation toward her interests. Although specific activities were often suggested by the professional, the participant determined whether or not these activities were undertaken and their length. That is, when the participant indicated she wanted to do something else, the activity was stopped. The active involvement of family members during the sessions offered a safe and positive environment, as well as an easier transfer of the learned social skills to the home environment.

The five equine-assisted sessions were recorded on video by the researcher to enable detailed observation and quantitative analysis of the participant’s behavior during the sessions. After the final session, the researcher evaluated the program with the parents, administered the SED-R [40], and asked both parents to fill out the SDQ [41,44] again. The study was conducted in accordance with the Declaration of Helsinki and was approved by the ethics committee of the host institution, after reviewing the information given to the participant’s parents and the informed consent form.

### 2.3. Measurements

Changes in the social and communication skills of the participant were studied by analyzing the questionnaires filled out by the parents and observing the video recordings.

#### 2.3.1. Scale for Emotional Development-Revised (SED-R)

The SED-R can be used to assess the social-emotional development of individuals with intellectual disabilities, and is increasingly used for people with ASD [40]. The social-emotional development of the participant was rated by means of a semi-structured interview of the researcher with two informants who know the individual well, in this case the parents. This was done by asking questions about the participant’s social skills across thirteen domains of emotional development: Dealing with your own body, Interacting with emotionally important others, Self-image in interaction with the environment, Dealing with a changing environment, Anxieties, Interacting with peers, Handling materials, Communication, Emotion differentiation, Aggression regulation, Play development, Moral development, and Emotion regulation. For each of these thirteen domains, the development of the participant was then rated by the researcher on an ordinal scale of development. This scale consists of five levels (phases), that range from a self-directed point of view to a full understanding of social rules and using appropriate social skills. The SED-R was administered before the sessions started and again after the final session.

#### 2.3.2. Strengths and Difficulties Questionnaire (SDQ)

The SDQ is a general screening instrument that can be used to assess both the psycho-social problems and strengths of children [41]. Items represent concrete (social) behaviors that parents rate on a three-point scale (not true, somewhat true, certainly true). The SDQ consists of 25 items, grouped into five scales: Emotional symptoms, Conduct problems, Hyperactivity/Inattention, Peer relationship problems, and Prosocial behavior. The total difficulties score can be calculated by adding the scores of the first four scales. In this study, the SDQ was filled out by both parents separately before the therapy sessions started, and again after the final therapy session.

#### 2.3.3. Video Observations

The video files were first separated into periods of relaxation exercises, horseback riding, and interaction games. Multiple periods with similar activities could occur within a single session (see Table 1). If a period lasted ten minutes or less, the participant’s behavior was observed during the full period. If a period lasted longer than ten minutes, the participant’s behavior during ten minutes in the exact middle of that period was observed. Adequate verbal communication and Tension were observed during riding and relaxation exercises. Turn-taking was observed during the interaction games. Separation anxiety and Emotions were observed during all periods.

A standardized coding scheme was constructed by two researchers, to make sure the videos were systematically observed. Coding proceeded in four rounds; each round was focused on specific behaviors. Before the observations started, the researcher who would code the videos was trained and inter-observer reliability measures were calculated for each round. A target percentage of 80% inter-observer agreement was considered to be sufficient. If this percentage was not reached immediately, the training and coding schemes were adjusted, after which inter-rater reliability was determined again by using a different video.

For Adequate verbal communication, we reached a final inter-rater agreement of 80%. Adequate verbal communication consisted of meaningful answers of the participant that matched the questions of the interaction partner, or relevant verbal initiations. We considered initiations of the participant relevant if they were focused on the current situation, if the participant shared information about herself, or if the participant asked relevant questions. We reached a final inter-rater agreement of 83% for Tension. We coded Tension when the participant showed muscle contractions in face, limbs or general posture, and when the participant showed repetitive body movements. For Turn-taking during interaction games we reached an inter-rater reliability of 100%. This coding scheme consisted of three codes. Inadequate turn-taking was coded when the participant inadequately responded when it was her turn, for example running away from others when it was actually her turn to tag, or when she did not respond at all. Adequate turn-taking with help was coded when the participant responded with the appropriate behaviors when it was her turn, but needed help to complete this task, such as tagging others together with an adult, or receiving additional instructions. The code Adequate turn-taking was used when the participant independently and accurately engaged in turn-taking.

The final inter-rater agreement for Separation anxiety and Emotions was 82%. We coded signs of Separation anxiety when the participant engaged in seeking close physical proximity to (one of) her parents, or verbally indicated the wish to do so, when this close physical proximity was not part of the activity, or not fitting the situation. We coded signs of Negative emotions when the participant cried, made loud noises or shouted. Positive emotions were coded when the participant was laughing or smiling, or verbally indicated happiness.

#### 2.3.4. Underlying Constructs

Table 2 lists the shared underlying constructs measured by the SED-R [40] and SDQ [41] subscales and the observations. In general, the SED-R covers a wider area than the other two instruments. The SED-R subscale Anxieties, for example, is only partly covered by the SDQ subscale Emotional problems (e.g., “Many worries or often seems worried”, “Many fears, easily scared”) and the observations of Separation anxiety. This also seems true for the SED-R subscale Dealing with own body and the observations of Tension, and the SED-R scale Play development and the observations of Turn-taking during interaction games. Despite a difference in name, the SED-R scale Moral development aligns with the SDQ scale Prosocial behavior (e.g., “Considerate of other people’s feelings”, “Helpful if someone is hurt”). The SED-R scale Aggression regulation shares its underlying construct with the SDQ Conduct problems scale (e.g., “Often has temper tantrums or hot tempers”).

### 2.4. Analysis

#### 2.4.1. Changes in Social and Communication Skills Reported by the Parents (RQ 1)

To answer the first research question, we calculated the scores on the SED-R and SDQ questionnaires administered to the parents before and after the intervention. We calculated the mean difference (post-test—pre-test) of each subscale. Differences between the SDQ scores of the mother and the father were compared by calculating percentages of agreement on the Total Difficulties score and the subscales for both the pre- and post-test (cf. [35]). To determine whether the results of the two instruments aligned, we compared similarities and differences between the SED-R and the SDQ questionnaire.

#### 2.4.2. Changes in Social and Communication Skills Observed during Sessions (RQ 2)

To answer the second research question about the social and communication skills observed during the sessions, we analyzed the development of the participant’s Adequate verbal communication, Tension, Separation anxiety, Turn-taking skills and Emotions over the course of the five sessions. The proportions of Adequate verbal communication, Tension, and Turn-taking were calculated by taking the duration of these observed behaviors in minutes divided by the total observed time in minutes. The analyses of Separation anxiety and Emotions (positive and negative) were based on the frequency of these observed behaviors divided by the total observed time in minutes (resulting in the frequency per minute).

#### 2.4.3. Similarities and Differences between Parental Report and Observations (RQ 3)

To answer the third research question, we compared the questionnaire and observation scores of similar constructs (see Table 2) to determine similarities and differences between the measures.

## 3. Results

### 3.1. Parental Report of Social and Communication Skills (RQ1)

#### 3.1.1. SED-R Questionnaire

Table 3 shows the results of the SED-R semi-structured interview that was held with both parents before and after the intervention. According to the parents, Self-image in interacting with the environment, Anxieties, Interacting with peers, Handling materials and Play development did not change. With regard to Interacting with emotionally important others, Dealing with a changing environment, Communication, Aggression regulation, and Moral development, the parents noticed an improvement of one level during the post-measurement. Lastly, the parents observed a bigger increase (two or three levels) with regard to the participant’s ability to Deal with her own body, Emotion differentiation and Emotion regulation.

The SED-R manual enables an interpretation of the scales and levels. On the pre-test, the parents indicated that the participant was still developing a sense of her own body (level 1) and was slowly learning to process sensory information and regulate her emotions (level 2). In addition, the participant did not have a proper sense of object permanence (Dealing with a changing environment, level 2). For six of the thirteen domains, the parents indicated that the participant scored on level 3. In this phase, children are still egocentric in their contact with others and in their emotions. This means, for example, that the participant could not engage in reciprocal conversations. With regard to her moral development, she needed clear guidelines and the presence of adults to obey these. The participant’s play development and handling of materials were well-developed (level 4) compared to her other social-emotional skills. This means that she was able to play with other children and could independently handle materials. On the post-test, all domains of social-emotional development were rated on level 3 or 4 by the parents. These levels are characterized by more independence and responsibility, taking initiative, and a growing sense of reality and empathy. The parents indicated that the participant was growing with regard to her sense of object permanence and seeing other people’s perspectives.

#### 3.1.2. SDQ Questionnaire

Table 4 shows the results of the SDQ questionnaire, filled out by the parents separately on the pre- and post-measurement. The participant’s mother reported a decrease of eight points on the total difficulties score, mostly caused by a decrease of Emotional problems and Peer relationship problems. In addition, the mother of the participant reported an increase of the participant’s Prosocial behavior with three points. The participant’s father indicated fewer problems (lower scores) and more Prosocial behavior on the pre-test compared to the participant’s mother. At the same time, he reported less improvement on the post-test. After the equine-assisted intervention, he reported a decrease of five points on the Total Difficulties score, mostly caused by a decrease of Peer relationship problems and a small decrease of Hyperactivity and inattention. According to the SDQ scoring instructions (2016), the Total Difficulties score given by the mother could be classified as “very high” on the pre-test, whereas the father’s score could be classified as “high”. On the post-test, both the Total Difficulties score given by the mother and father could be classified as “slightly raised”.

In general, the mother of the participant reported more problems on the pre-test and a bigger decrease on the post-test. The scores of the parents on the Total Difficulties score aligned for 79.2% on the pre-test and 87.5% on the post-test. Parents also agreed with regard to their scores on the subscales Conduct problems (100% agreement on both the pre- and post-test), Hyperactivity/inattention (85.7% agreement on the pre-test and 83.3% on the post-test) and Peer relationship problems (100% agreement on the pre-test, 80% on the post-test). The parents did not agree in their scores on Emotional problems (50% agreement) and Prosocial behavior (62.5% agreement) on the pre-test, but they did agree on the post-test (100%). In total, the average agreement for all subscales together was 79.6% on the pre-test and 91.8% on the post-test.

#### 3.1.3. Similarities and Discrepancies between the SED-R and SDQ Questionnaires

The mother of the participant reported a decrease on the SDQ scale Emotional symptoms after the equine-assisted intervention. This seemed in line with the increase on the SED-R subscales Emotion differentiation and Emotion regulation. The mother of the participant also reported higher scores on the SDQ scale Prosocial behavior after the intervention, which seemed in line with the increase on the SED-R scale Moral development. Both parents reported no difference on the SDQ subscale Conduct problems after the intervention, but they did report an increase in Aggression regulation on the SED-R questionnaire. Parents also reported less Peer relationship problems on the SDQ after the intervention, while the SED-R score for Interacting with peers stayed similar.

### 3.2. Observations of Social and Communication Skills during the Therapy (RQ 2)

**Figure 1:**
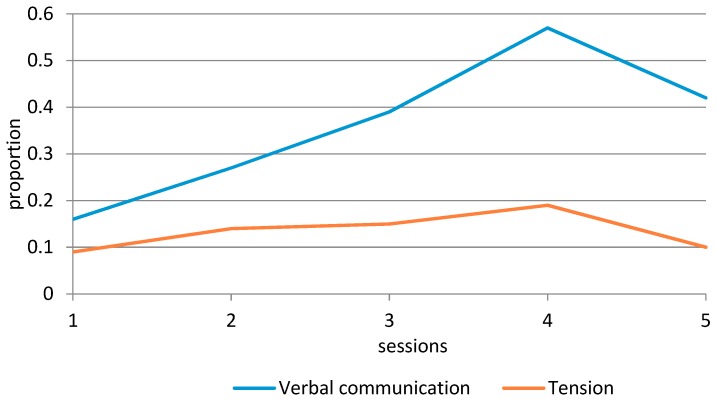
Graphical display of the participant’s verbal communication and tension over time.

**Figure 2:**
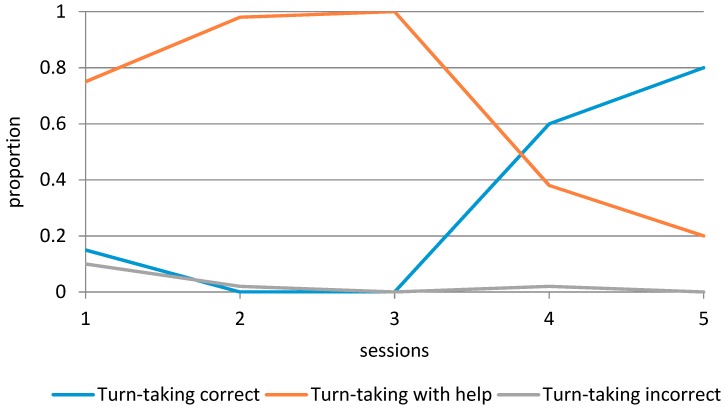
Graphical display of the participant’s turn-taking skills over time.

**Figure 3:**
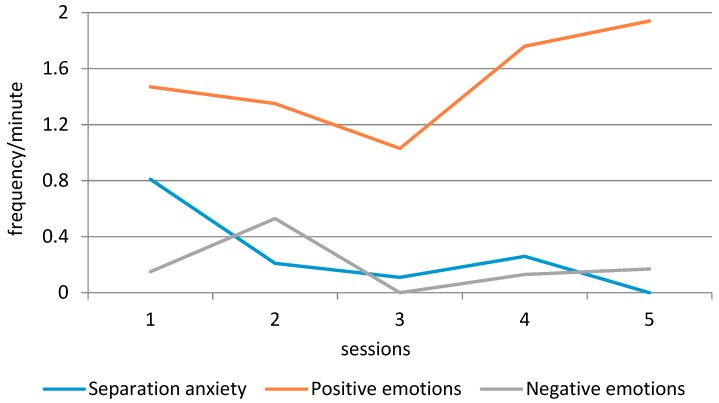
Graphical display of the participant’s separation anxiety and emotions over time.

An overview of the social and communication skills observed during the therapy can be found in Table 5 and Figure 1, Figure 2 and Figure 3. Adequate verbal communication increased steadily from the first session (16% of the time) to the fourth session (57% of the time), and then decreased slightly to the level of the third session (42% of the time). The observed Tension of the participant initially increased over time (9%–19%), but then decreased to the level of the first session (10%). The Turn-taking skills of the participant showed an interesting development (see Figure 2). Correct turn-taking, which was uncommon during the first three sessions, showed a considerable increase in the fourth session (60% of the time), while Turn-taking with help decreased to 38% of the time. Separation anxiety initially decreased until the third session (from 0.81 to 0.11 times per minute), then increased in the fourth session (0.26), and was completely absent during the fifth session. The Positive emotions of the participant decreased from 1.47 to 1.03 times per minute in the first three sessions, but then increased in the fourth and fifth session (1.76 and 1.94 times per minute). Negative emotions increased from 0.15 to 0.53 times per minute, were completely absent during the third session, and then increased again to 0.13 and 0.17 times per minute in the last two sessions.

In general, most observed behaviors improved over time, and the fourth visit seemed a turning point, as evidenced by a considerable increase in Adequate verbal communication, Positive emotions, and Correct turn-taking. Interestingly, we also observed higher scores for Tension and Separation anxiety during session 4, and small increases in Negative emotions and Incorrect turn-taking.

### 3.3. Similarities and Discrepancies between the Questionnaires and Observations (RQ 3)

The SED-R Communication scores improved after the equine-assisted intervention, which was also visible in the increase of Adequate verbal communication observed during the sessions. With regard to Emotions, the parents of the participant indicated increased Emotion differentiation and Emotion regulation on the SED-R, and less Emotional problems on the SDQ after the equine-assisted intervention. This was reflected in an observed decrease of Negative emotions and increase of Positive emotions during the sessions. Interestingly, parents reported no positive or negative changes in Play development on the SED-R, whereas the Turn-taking skills of the participant during play showed a considerable development over the course of five sessions. Lastly, while the SED-R scores showed no change in Anxieties, the observations did show a decrease in Separation anxiety over the five sessions.

## 4. Discussion

In this study we compared the results of several measures in the context of an equine-assisted intervention for a child (female, eight years old) with Autism Spectrum Disorder (ASD). We conducted a semi-structured interview with the parents of the participant before and after the intervention (Scale for Emotional Development-Revised (SED-R; [40]), and administered the Strengths and Difficulties questionnaire (SDQ; [41]) to both parents. In addition, we systematically observed the social and communication skills of the participant during five equine-assisted sessions.

Parents reported an improvement of their daughter’s social and communication skills on both the SED-R and SDQ. With regard to the SED-R scores, the biggest improvement was reported for the participant’s ability to deal with her own body, emotion differentiation and emotion regulation. With regard to the SDQ scores, the participant’s improvement in peer relationships and prosocial behavior stood out. The parents agreed for 79.2% on the SDQ administered before the intervention, and for 87.5% on the SDQ administered after the intervention. The mother reported more problems on the pre-test and a bigger improvement. The SDQ scores for emotional symptoms given by the mother aligned with the SED-R scores on emotion differentiation and emotion regulation. In addition, the mother’s scores on the SDQ subscale prosocial behavior aligned with the SED-R scores on moral development. No relationship between the father’s SDQ scores and the SED-R scores could be detected. A discrepancy between the two instruments was found for conduct problems and peer relationships.

In general, the systematic observations of the participant’s behavior during the sessions showed an improvement over time, apart from Tension, which exhibited similar levels during the first and final sessions. Across all measures, the fourth visit seemed a turning point, as evidenced by a considerable increase in positive behaviors, but also higher scores for tension and separation anxiety, and small increases in negative emotions and incorrect turn-taking.

With regard to adequate verbal communication, the SED-R scores aligned with the observations during the sessions. For emotion regulation and differentiation, the scores on the SED-R, SDQ and observations aligned. While no changes in play development were reported on the SED-R, improvement in a specific aspect of play, turn-taking, was observed during the sessions. Lastly, while the SED-R scores showed no change in anxieties, the observations did show a decrease in separation anxiety during the five sessions.

In light of our research aim to compare and contrast these measures, and thereby to assess their applicability in the context of animal-assisted interventions (AAI), we first discuss the difference between the two questionnaires. The SDQ can be considered as a more general screening instrument [41], while the SED-R is specifically focused on children with disabilities, such as ASD [40]. Although there are differences between the outcomes of these two instruments, the results do not show that one of these provided a more positive view of the participant’s development over time than the other. The SED-R results indicated that the participant learned to better regulate her aggression over the course of the intervention, which was in contrast with the SDQ results. For peer relationships, the SDQ scores after the intervention provided a more positive view. With regard to the similarities between the questionnaire results, note that only the mother’s scores on the SDQ scales Emotional symptoms and Prosocial behavior aligned with the SED-R scores.

This brings us to the second point, namely the difference between the two parents who filled out the SDQ before and after the intervention (note that the SED-R is a semi-structured interview, administered to both parents at the same time). The agreement between the parents was moderate to high, yet, the mother reported more problems on the pre-test and a bigger improvement on the post-test. She scored considerably higher on the subscale Emotional problems compared to the father. Earlier research has shown that mothers report internalizing problems more often than fathers or other informants [33,35]. Interestingly, the SDQ scales on which the parents agreed most, Conduct problems and Peer relationship problems, were not in line with the SED-R scores on the subscales Aggression regulation and Interacting with peers. A possible reason for the discrepancy might be that while the problems in social contact declined, the participant still needed to develop skills to truly engage in reciprocal interactions with others. Similarly, the participant might have improved in regulating aggression, while a single specific conduct problem still existed.

The third difference that can be discussed, and that is especially valuable in the AAI context, is the difference between the interpretation of the questionnaire and the observation data. While questionnaires provide an idea of how the behavior of the child is experienced by the parents, the advantage of observational methods is that they capture changes in children’s social interaction and communication skills in a natural context [36,37]. Although some of the observations were similar to the questionnaire results, there were also some differences. First, we saw a considerable positive difference in a specific social skill during play, turn-taking, while no change in play development was reported on the SED-R. Note that the parents already scored quite high on the SED-R before the equine-assisted intervention, by which they indicated that the participant had a growing sense of other people’s perspective and reality. However, the observations show that the participant needed considerable help in turn-taking during the first sessions, which requires the understanding of other people’s perspective. It could be that the parents did not consider this specific aspect of play when responding to the SED-R questions, for example because they did not engage in rule-based games with their daughter outside the therapy context. Another possibility is that the participant is able to take turns accurately, but that it takes a while for her to adopt a new role when the game changes. Indeed, other studies have found differences in attention between children with ASD and typically developing children [46]. The topic of turn-taking has recently caught the attention of researchers in the AAI context. Researchers have suggested that the practice of turn-taking skills is valuable for children with ASD and should occur in a setting that is positive and relatively stress-free. Some authors argue that AAI provides such a context [47]. A recent study of Griffioen and colleagues [48] shows that AAI can positively influence the turn-taking skills of children with ASD, although the results depend on the child’s ability to verbally express him/herself.

A second difference between the observations and questionnaires was found between the SED-R subscale Anxieties (no change) and the observations of Separation anxiety, which showed a considerable improvement over the five equine-assisted sessions. While one could argue that separation anxiety is a very specific type of anxiety that does not fully cover the SED-R subscale, note that the parents indicated this as a pressing issue during the intake session. This discrepancy between the parental report and observations might be due to the fact that the observations were limited to the equine-assisted sessions. In other words, it is possible that the participant still showed separation anxiety in other contexts, which the parents took as a reference. Researchers have suggested that people can form an attachment to animals that is of the same quality as the caregiver–child attachment [49]. Because of this bond, children feel safe in the AAI context and can further explore their social skills [50]. Given this view and given that separation anxiety occurs when being separated from an attachment figure [42], we could infer, albeit cautiously, that the presence of the horse may have had a calming effect on the participant. That said, we ultimately would like to see a transfer of the behavioral change in the AAI context to daily life. The results on the SED-R seem to suggest that this was not the case for our participant, although our research design was limited by only observing the participant in the AAI context.

There is currently a call for randomized controlled trials in the field of AAI. While such studies are challenging in terms of randomly allocating subjects to conditions and blinding the participants and/or the research team, they also require considerable sample sizes. Although large samples are clearly an advantage to further strengthen the empirical base of AAI, the downside is that taking reliable observations with these sample sizes becomes almost impossible, as these are quite time-consuming. We therefore advocate the use of observations on subsamples, as these can be used to further investigate the mechanisms of AAI.

To give an example of such a hypothesis, there is currently a dose–response discussion in the field of AAI. Multiple researchers have noticed that we do not know the minimum number of AAI sessions needed to see improvement in the participant’s behavior or skills [16,51,52]. Although the current study is a single-subject design, from which we cannot draw definite conclusions, our study has generated an important hypothesis in this regard for children with ASD. The fourth visit seemed a turning point, as evidenced by a considerable increase in adequate verbal communication, positive emotions, and correct turn-taking. Interestingly, we also observed higher scores for tension and separation anxiety during session 4, and small increases in negative emotions and incorrect turn-taking. After session 4, these “negative” behaviors improved considerably. Using questionnaires only, we would have missed this possible turning point.

## 5. Conclusions

In this study we compared the results of several measures in the context of an equine-assisted intervention for a child (female, eight years old) with Autism Spectrum Disorder (ASD). Before and after the equine-assisted sessions we conducted a semi-structured interview with the participant’s parents, asked parents to fill out a general screening instrument separately, and observed the participant’s social and communication skills during five equine-assisted sessions. Although all instruments indicated an improvement of the participant’s social and communication skills, differences were found between the interview and questionnaires with regard to parents’ perception of aggression regulation and interacting with peers. Differences with regard to parental report and observations were found for play development and anxiety. The observations provided a detailed view of the child’s development during the equine-assisted sessions. However, because the observations in this study were limited to the AAI context, it is hard to determine whether or not the behavioral improvements generalized to other situations. Yet, the observations also provided us with an important hypothesis about the number of AAI sessions needed for children with ASD, which can be further explored in future research. We therefore call for the use of observations on (sub)samples in future research, as these can be used to further investigate the mechanisms of AAI.

## Figures and Tables

**Table 1:** Example of observation periods and behaviors during session 1.

Periods	Total Duration	Observation Period	Observed Behaviors
Horseback riding	05.24–36.36	16.00–26.00	Verbal communication Tension Separation anxiety Emotions
Interaction game	38.16–40.40	38.16–40.40	Turn-taking Separation anxiety Emotions
Interaction game	44.10–55.44	45.00–55.00	Turn-taking Separation anxiety Emotions
Horseback riding	57.16–1.04.16	57.16–1.04.16	Verbal communication Tension Separation anxiety Emotions
Interaction game	1.24.04–1.28.13	1.24.04–1.28.13	Turn-taking Separation anxiety Emotions
Relaxation exercise	1.31.35–1.32.54	1.31.35–1.32.54	Verbal communication Tension Separation anxiety Emotions
Interaction game	1.33.20–1.38.35	1.33.20–1.38.35	Turn-taking Separation anxiety Emotions

**Table 2:** Shared underlying constructs measured by the two questionnaires administered to the parents and systematic observations by the researchers.

Scale for Emotional Development-Revised (SED-R) Subscale (Parents)	Strength and Difficulties Questionnaire (SDQ) Subscale (Parents)	Observations (Researchers)
Self-image in interacting with environment		
Dealing with own body ^1^		Tension
Interacting with emotionally important others		
Self-image in interacting with environment		
Dealing with a changing environment		
Anxieties ^1^	Emotional problems	Separation anxiety
Interacting with peers	Peer relationship problems	
Handling materials		
Communication		Adequate verbal communication
Emotion differentiation	Emotional problems	Emotions
Aggression regulation	Conduct problems	
Play development ^1^		Turn-taking in games
Moral development	Prosocial behavior	
Emotion regulation	Emotional problems	Emotions

^1^ The constructs underlying the SED-R subscales Dealing with own body, Anxieties and Play development are partly covered by the observations of respectively Tension, Separation anxiety and Turn-taking in games. The SED-R covers a wider area compared to the other two instruments. The SDQ Hyperactivity subscale (and the Total Difficulties score) are not listed in the table, as their underlying constructs are not sufficiently covered by the other two instruments.

**Table 3:** Results of the Scale for Emotional Development-Revised (SED-R), administered before and after the intervention.

Domain	Pre-Test	Post-Test	Difference
Dealing with own body	1	4	3
Interacting with emotionally important others	3	4	1
Self-image in interacting with environment	3	3	0
Dealing with a changing environment	2	3	1
Anxieties	3	3	0
Interacting with peers	3	3	0
Handling materials	4	4	0
Communication	3	4	1
Emotion differentiation	2	4	2
Aggression regulation	2	3	1
Play development	4	4	0
Moral development	3	4	1
Emotion regulation	2	4	2

**Table 4:** Results of the SDQ, administered before and after the equine-assisted therapy.

Scale	T_0_ Mother	T_1_ Mother	T_0_ Father	T_1_ Father
Emotional symptoms	8	4	4	4
Conduct problems	1	1	1	1
Hyperactivity/Inattention	7	6	6	5
Peer relationship problems	8	5	8	4
Prosocial behavior	5	8	8	8
Total difficulties score	24	16	19	14

**Table 5:** Observations of social and communication skills during the therapy sessions.

Session	Verbal Communication	Tension	Turn-Taking Correct	Turn-Taking with Help	Turn-Taking Incorrect	Separation Anxiety	Positive Emotion	Negative Emotion
1	0.16	0.09	0.15	0.75	0.10	0.81	1.47	0.15
2	0.27	0.14	0.00	0.98	0.02	0.21	1.35	0.53
3	0.39	0.15	0.00	1.00	0.00	0.11	1.03	0.00
4	0.57	0.19	0.60	0.38	0.02	0.26	1.76	0.13
5	0.42	0.10	0.80	0.20	0.00	0.00	1.94	0.17
